# Effectiveness of providing university students with a mindfulness-based intervention to increase resilience to stress: 1-year follow-up of a pragmatic randomised controlled trial

**DOI:** 10.1136/jech-2020-214390

**Published:** 2020-09-10

**Authors:** Julieta Galante, Jan Stochl, Géraldine Dufour, Maris Vainre, Adam Peter Wagner, Peter Brian Jones

**Affiliations:** 1 Department of Psychiatry, University of Cambridge, Cambridge, UK; 2 National Institute for Health Research Applied Research Collaboration East of England, Cambridge, UK; 3 Department of Kinanthropology, Charles University, Prague, Czech Republic; 4 University Counselling Service, University of Cambridge, Cambridge, UK; 5 Universities and Colleges Division, British Association for Counselling and Psychotherapy, Lutterworth, UK; 6 MRC Cognition and Brain Sciences Unit, University of Cambridge, Cambridge, UK; 7 National Institute for Health Research Collaboration for Leadership in Applied Health Research and Care East of England, Cambridge, UK; 8 Norwich Medical School, University of East Anglia, Norwich, UK

**Keywords:** Randomised trials, Mental health, Psychological stress, Health services

## Abstract

**Background:**

There is concern that increasing demand for student mental health services reflects deteriorating student well-being. We designed a pragmatic, parallel, single-blinded randomised controlled trial hypothesising that providing mindfulness courses to university students would promote their resilience to stress up to a year later. Here we present 1-year follow-up outcomes.

**Methods:**

University of Cambridge students without severe mental illness or crisis were randomised (1:1, remote software-generated random numbers), to join an 8-week mindfulness course adapted for university students (Mindfulness Skills for Students (MSS)), or to mental health support as usual (SAU).

**Results:**

We randomised 616 students; 53% completed the 1-year follow-up questionnaire. Self-reported psychological distress and mental well-being improved in the MSS arm for up to 1 year compared to SAU (p<0.001). Effects were smaller than during the examination period. No significant differences between arms were detected in the use of University Counselling Service and other support resources, but there was a trend for MSS participants having milder needs. There were no differences in students’ workload management; MSS participants made more donations. Home practice had positive dose–response effects; few participants meditated. No adverse effects related to self-harm, suicidality or harm to others were detected.

**Conclusion:**

Loss to follow-up is a limitation, but evidence suggests beneficial effects on students’ average psychological distress that last for at least a year. Effects are on average larger at stressful times, consistent with the hypothesis that this type of mindfulness training increases resilience to stress.

**Trial registration number:**

ACTRN12615001160527.

## INTRODUCTION

Official statistics show that the prevalence of mental health disorders among children and young people in England, emotional disorders in particular, has been increasing over time, reaching almost one in five aged 17–19 years in 2017.^1^ In England, now over 50% of young people enrol in higher education institutions,^2^ which have a golden yet under-used opportunity for prevention of mental illness in young people.^3 4^ This seems particularly relevant as there are concerns that the pressure that young people experience when they transition to university can contribute to mental health issues for some of them.^5^ While more research is needed,^6^ it is clear that the number of university students accessing counselling services has increased faster than the growth in student numbers.^7^

Mindfulness, a non-stigmatising means of training the attention for the purpose of mental health promotion, has become popular in universities.^8^ In this context, mindfulness practice is often defined as learning to pay attention to what is happening in the present moment in the mind, body and external environment with an attitude of curiosity and kindness.^9^ There is evidence for its effectiveness in preventing psychological distress,^10^ and improving symptoms of common mental disorders.^11^

In 2016, we completed the Mindful Student Study, a randomised controlled trial (RCT) to confirm the effectiveness of a preventative mindfulness-based programme tailored to university students called Mindfulness Skills for Students (MSS).^12^ In a recent publication, we confirmed our primary hypothesis that MSS would reduce students’ psychological distress during the examination period (3–6 months after randomisation) compared with access to mental health support as usual (SAU).^13^ A reduction in distress under exam conditions was deemed an indicator of resilience to stress. These results are consistent with other evidence, although data on longer-term effects and on use of mental health services are sparse.^10^

Participants in the Mindful Student Study were followed up for a year post randomisation. Outcomes pertaining to this time point and participants’ trajectories are presented herein. Consistent with the idea of resilience and prior evidence, our main hypothesis for this analysis was that MSS would have a long-term effect on psychological distress still outperforming SAU for reducing psychological distress after 1 year, but that this effect would be smaller than that during the examination period because students would no longer be under the examination universal stressor.

## METHODS

The Cambridge Psychology Research Ethics Committee approved the trial on 25 August 2015 (PRE.2015.060). This research conforms to the principles embodied in the Declaration of Helsinki. The protocol^12^ was submitted to the Australian New Zealand Clinical Trials Registry on 31 August 2015, before the study began, and accepted on 30 October 2015 (trial registration: ACTRN12615001160527).

### Randomisation and blinding

We conducted a pragmatic RCT with two parallel arms and a one-to-one allocation ratio testing the superiority of mindfulness training provision compared with no provision. All the students at the University of Cambridge were invited to join the study. Those who responded positively were randomised via remote survey software (Qualtrics, concealed from researchers) using computer-generated random numbers (simple randomisation) to being offered the MSS course plus SAU, or to SAU alone. Participants were aware of group allocation.

We set up an independent data monitoring and ethics committee (IDMEC), and co-produced the trial with stakeholders. Further details including sample size calculations can be found in previous publications.^12 13^

### Eligibility

Eligibility criteria were assessed by participants themselves, and based on those used routinely by the University of Cambridge Counselling Service (UCS) for the MSS courses. Inclusion criteria were as follows: (a) current undergraduate or postgraduate students at the University of Cambridge; (b) who believed they could attend at least seven sessions of the course. Exclusion criteria were as follows: (a) currently suffering from severe periods of anxiety or depression; (b) experiencing severe mental illness such as hypomania or psychotic episodes; (c) recent bereavement or major loss; and (d) experiencing any other serious mental or physical health problem that would affect their ability to engage with the course.

Two cohorts of students were recruited (October 2015 and January 2016; no main outcome differences were found between cohorts).^13^ MSS courses were free to students. A total of £11 was available to each participant as a token of appreciation for questionnaire completion.

### Intervention

The MSS intervention consisted of a secular, face-to-face, group-based skills training programme based on the course book ‘Mindfulness: A Practical Guide to Finding Peace in a Frantic World’,^14^ and adapted for university students. This course aimed to optimise well-being and resilience for all students, and was not specifically developed for those with distress in a clinical range. Seven MSS courses ran in parallel during university terms, with up to 30 students in each course, all delivered by an experienced and certified mindfulness teacher. The eight, weekly sessions lasted 75–90 min. Sessions included mindfulness meditation exercises, periods of reflection and inquiry, and interactive exercises. Students were encouraged to also practise at home and were given reading materials. The recommended home practice time started at 8 min, then increasing to 15–25 min/day. It included guided formal meditations (from here on: ‘formal practice’) and other practices such as a mindful walking and mindful eating (from here on: ‘informal practice’). Students were contacted by email when they missed a session to check whether the absence related to a negative experience with mindfulness. Students were also given the opportunity to talk with the teacher in confidence outside course times. Further details can be found in previous publications.^12 13^

SAU consisted of access to comprehensive centralised support at the UCS in addition to support available from the university and its colleges, and from health services including the National Health Service, external to the University. Participants randomised to SAU were guaranteed a space in the following year’s mindfulness courses and were requested to inform the team if they decided to learn mindfulness elsewhere during the follow-up period.

### Measures

Self-reported data were collected using online questionnaires accessed by participants via a unique link. The examination period as defined by the Student Registry spanned 16 May 2016–10 June 2016, the most stressful weeks of the academic year for most students (not all have exams, approximately 14% did not in our sample), approximately 6 months after randomisation for Cohort 1, and 3 months after randomisation for Cohort 2. Online supplemental table 1 lists all trial outcome measures and data collection time points.

**Table 1:** One-year follow-up psychological distress (CORE-OM and its subscales) and well-being (WEMWBS) outcomes

		All	MSS	SAU
CORE-OM total mean score	N	338	169	169
	Mean	0.86	0.80	0.93
	SD	0.52	0.49	0.55
	Median	0.74	0.68	0.82
	Min–Max	0–2.76	0–2.76	0–2.68
CORE-OM well-being subscale mean score	N	338	169	169
	Mean	1.04	0.98	1.10
	SD	0.74	0.73	0.75
	Median	1	0.75	1
	Min–Max	0–3.50	0–3.50	0–3.50
CORE-OM symptoms subscale mean score	N	337	168	169
	Mean	1.13	1.06	1.20
	SD	0.71	0.68	0.75
	Median	1	0.92	1.08
	Min–Max	0–3.58	0–3.33	0–3.58
CORE-OM functioning sub-scale mean score	N	335	168	167
	Mean	0.92	0.85	0.99
	SD	0.57	0.55	0.59
	Median	0.83	0.75	0.92
	Min–Max	0–3.17	0–3.17	0–2.83
CORE-OM risk subscale mean score	N	339	179	169
	Mean	0.08	0.06	0.10
	SD	0.21	0.17	0.25
	Median	0	0	0
	Min–Max	0–1.17	0–1.17	0–1.17
WEMWBS total score	N	335	168	167
	Mean	49.92	51.06	48.77
	SD	9.31	9.58	8.92
	Median	51	52	50
	Min–Max	17–70	17–70	25–70

CORE-OM, Clinical Outcomes in Routine Evaluation Outcome Measure; MSS, Mindfulness Skills for Students; Min–Max, minimum and maximum values; SAU, support as usual; WEMWBS, Warwick-Edinburgh Mental Wellbeing Scale.

10.1136/jech-2020-214390.supp1 Supplementary data

#### Self-reported mental health

Psychological distress was measured with the Clinical Outcomes in Routine Evaluation Outcome Measure (CORE-OM), a 34-item scale that has been widely used with UK university students.^15^ Higher scores mean more distress. The total mean score (range 0–4) is obtained by dividing the total score by the number of completed items (as long as no more than three items have been missed).^16^ This measure also contains four subscales: subjective well-being (4 items), problems/symptoms (12 items), life functioning (12 items) and risk/harm (6 items). We have primarily used the full-scale total mean score, but also explored the sub-scale mean scores to see whether the effect of mindfulness would focus on specific dimensions of distress.

Mental well-being was assessed with the 14-item Warwick-Edinburgh Mental Wellbeing Scale (WEMWBS).^17^ The total score is calculated by adding the response values of all items (range 14–70, higher scores indicate greater well-being).

#### Use of mental health support resources

Following confidentiality protocols, the UCS provided the research team with information about which participants used their services, what type of services they used and how frequently they were used. The UCS offers a variety of support services for students depending on their needs and ranging from workshops or therapy groups, to attending a consultation with a counsellor, CBT therapist, mental health advisor or sexual assault & harassment advisor. We assessed usage of the services from the moment each participant was randomised up to a year after that, and usage during the examination period specifically. We also assessed UCS services according to the intensity of support. For this, blind to any data and before analysis, GD (accredited senior psychotherapist and head of service) and three accredited senior counsellors categorised services according to the intensity of the support they provide into low, medium or high, reflecting the severity of the mental health problems that they are intended to address (online supplemental table 2). Then, these categories were uniformly applied to the type of service variable in the data set provided by the UCS.

**Table 2:** One-year follow-up and cumulative results for various outcome measures

		All		MSS		SAU	
Use of UCS services	Participants who used the UCS during the full follow-up period	122	20%	57	18%	65	21%
(nMSS=309, nSAU=307)	Total number of contacts	517		238		279	
	Number of contacts per user among users (median range)	3	19	3	17	3	19
	Participants who used the UCS during the exam period	32	5%	13	4%	19	6%
Severity of UCS contacts	Total number of low severity contacts	49	9%	29	12%	20	7%
(nMSS=309, nSAU=307)	Total number of medium severity contacts	449	87%	206	87%	243	87%
	Total number of high severity contacts	19	4%	3	1%	16	6%
Mental health resources used (self-report)	None	162	49%	78	47%	84	51%
(nMSS=166, nSAU=165)	Supervisor/director of studies/tutor	91	27%	44	27%	47	28%
	UCS counsellor/mental health advisor	66	20%	31	19%	35	21%
	College nurse/counsellor	60	18%	34	20%	26	16%
	GP	57	17%	26	16%	31	19%
	External professional counsellor/psychotherapist/psychologist	40	12%	22	13%	18	11%
	Psychiatrist	19	6%	10	6%	9	5%
	Other	16	5%	12	7%	4	2%
	Chaplain	15	5%	8	5%	7	4%
	Complementary medicine	14	4%	5	3%	9	5%
	Helpline, nightline, Samaritans	7	2%	4	2%	3	2%
	Emergency services	3	1%	1	1%	2	1%
	Used any resource	169	51%	88	53%	81	49%
	Number of resources per user among users (median range)	2	8	2	8	2	7
Workload perceived as manageable	Definitely agree	51	15%	30	18%	21	13%
(nMSS=165, nSAU=166)	Mostly agree	136	41%	66	40%	70	42%
	Neither agree nor disagree	51	15%	20	12%	31	19%
	Mostly disagree	68	21%	37	22%	31	19%
	Definitely disagree	25	8%	12	7%	13	8%
Adverse events	Participants with adverse events between exam period and 1-year follow-up time points	11	2%	4	1%	7	2%
(nMSS=179, nSAU=169)	One-year cumulative count of adverse events	60		28		32	
Altruism	Participants donating at 1-year follow-up	191	57%	109	65%	82	49%
(nMSS=168, nSAU=167)	One-year cumulative count of donations	679		403		276	

Showing n(%) unless otherwise stated.

GP, general practitioner; MSS, Mindfulness Skills for Students; SAU, support as usual; UCS, University Counselling Service.

To assess the use of the wider range of mental health support resources, participants were asked ‘Have you turned to any of the following resources to discuss your mental health during the past year?’, and a list of available resources was presented to them. They could choose multiple items and there was an ‘other resources’ option with a text box to specify any unlisted resources. We analysed the usage of resources overall and by type.

#### Other outcomes compared between arms

Mindfulness aims to cultivate a general attitude of care and kindness, prompting claims, and some evidence, that it may also increase altruistic behaviour.^18^ We therefore incorporated an opportunistic measure of altruism, based on offering high street shopping vouchers to participants upon questionnaire completion (equivalent to £3 at post-intervention and 1-year follow-up, and £5 during the examination period) with a choice to donate them to a named charity.

We have also measured perceived university course workload. This was assessed by asking participants to indicate agreement on a five-point Likert scale with the statement ‘The workload on my course was manageable during the past year’.

We report the number of adverse scores recorded at the 1-year follow-up (identified by CORE-OM risk subscales above standard thresholds). Such ratings were defined as adverse events not necessarily caused by the intervention (as opposed to adverse effects, which would be). For further detail, see the trial protocol.^12^

#### Mindfulness practice effects

In order to assess mindfulness practice dose–response effects, we monitored participants’ practice throughout the follow-up. Within the MSS arm, formal and informal practice were self-reported via two questions asked at each time point except for baseline (eg, ‘During the mindfulness course did you practice mindfulness informally at home (eg, mindful living, mindful walks, mindful pauses, mindful attitudes)?’, ‘Have you been practising mindfulness formally (meditation practice) since you finished your mindfulness course?’). Attendance at mindfulness courses was registered. Also, at each time point, SAU participants were asked whether they had practised meditation elsewhere (eg, ‘About how many hours have you spent meditating in total since May, when we last sent you a questionnaire?’) and the type of meditation practised.

### Statistical methods

All analyses were conducted according to intention-to-treat at an alpha level of p=0.05 (two-sided). Logistic regression was employed to assess baseline predictors of outcome completeness using R version 3.4.4.^19^

The expected average trajectory for each arm over time on psychological distress and well-being was estimated using latent growth curve modelling,^20 21^ controlling for cohort, gender and age (variables controlled for in the primary outcome analysis as pre-specified in the protocol).^12 13^ Multiple imputation was not employed.

For comparing differences between arms in the proportion of users of UCS and other support resources, we used χ^2^ tests. Differences in the number of UCS contacts per user, or number of support resources, were compared using quasi-Poisson regression.

We used a hierarchical multinomial logit model in MPlus to compare differences between arms in terms of intensity of service use provision.^22^ This accounts for the hierarchical nature of the data structure, as any one student can use any particular service one or more times, and services belong to different levels of intensity. We expressed results as ORs. We also used χ^2^, quasi-Poisson regression and ORs to compare altruism and workload by arm.

To assess dose–response effects of mindfulness practice on psychological distress (the trial’s main outcome), the basic growth model mentioned earlier was extended with time-varying covariates representing mindfulness practice and distress reported at each time point. One model was created to assess formal mindfulness meditation, and another to assess informal mindfulness practice. These models also controlled for cohort, gender and age. Mindfulness practice data required pre-processing to include within the models (see online supplemental materials for detail).

## RESULTS

One-year follow-up questionnaire data were collected between 26 September 2016 and 11 October 2016 for Cohort 1, and between 10 Jamuary 2017 and 23 Jamuary 2017 for Cohort 2. Of the 616 randomised participants (MSS=309, SAU=307), 326 (53%) completed the 1-year follow-up questionnaire (MSS=161, 52%, SAU=165, 54%, online supplemental figure 1). No reasons were given for non-completion. There were no significant baseline differences between completers and non-completers, except that completers were less likely to be final year students. This may be explained by the fact that those who were in their final year at the beginning of the study may no longer have had the university email account used to contact them 1 year later (nor was a non-university address shared when requested ahead of their departure). Leaving university might have also reduced investment in the study.

**Figure 1:**
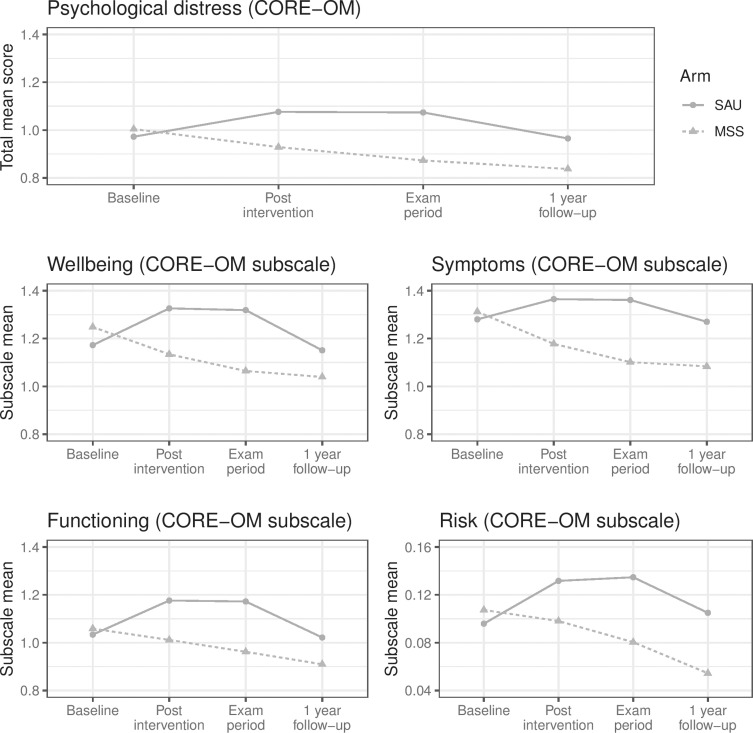
Multiple group growth model trajectories for psychological distress outcome (CORE-OM total mean and its subscales: well-being, symptoms, functioning and risk). CORE-OM, Clinical Outcomes in Routine Evaluation Outcome Measure; MSS, Mindfulness Skills for Students; SAU, support as usual.

### Self-reported mental health

Table 1 shows CORE-OM total mean scores and subscale mean scores, overall and by arm measured at 1-year follow-up. Average distress levels were lower at this time point than at any previous ones.^13^ To evaluate the long-term effect of mindfulness training, we have parameterised the growth model (online supplemental figure 2) such that the slope estimate can be interpreted as the difference in CORE-OM total mean scores between arm trajectories at the 1-year follow-up adjusted for our a priori set of baseline covariates. This slope takes the value of −0.22 (SE=0.05, p<0.001) suggesting that the MSS course reduces psychological distress for at least 1 year compared to SAU. This reduction is slightly smaller than that during the examination period (−0.25 points).^13^

**Figure 2:**
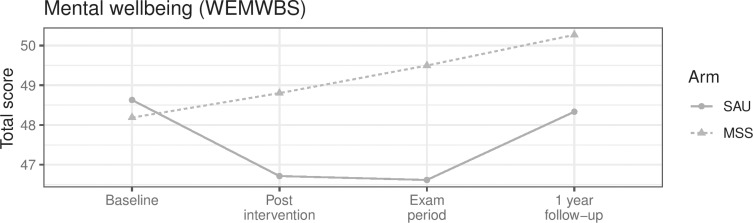
Multiple group growth model trajectories for well-being outcome (WEMWBS). WEMWBS, Warwick-Edinburgh Mental Wellbeing Scale; MSS, Mindfulness Skills for Students; SAU, support as usual.

Figure 1 shows the estimated trajectory by arm including CORE-OM subscales. The trajectory of the MSS group CORE-OM total mean score is an inverted U-shaped curve: the differences with the SAU group are larger at mid-follow-up time points. Subscales show very similar patterns to the total mean score.

Table 1 shows WEMWBS total scores overall and by arm measured at 1-year follow-up. Average well-being levels were higher at this time point than at any previous ones.^13^ The latent growth model, built in the same way as that for CORE-OM, shows that the difference in total WEMWBS scores between SAU and MSS was 2.73 (SE=1.03, p=0.008). This suggests that the MSS course improves well-being for at least 1 year compared to SAU, although the difference with SAU lies slightly below the ‘minimum detectable change’ for this instrument (defined as 3 points^23^). Figure 2 shows the modelled trajectory by arm.

### Use of mental health support resources

Table 2 shows UCS service usage overall and by arm. Overall, 20% of all the study participants (122 of 616) used (ie, attended) at least one of the services offered by the UCS during the full follow-up year, 5% during the examination period. Many UCS users had more than one contact with the UCS (median of three contacts among those who used the UCS). Sixteen participants booked UCS services but did not attend. No significant differences between arms were detected in the proportion of UCS users (χ^2^=0.56, df=1, p=0.46) or in the number of contacts per user (quasi-Poisson regression coefficient= −0.17, p=0.46). Restricting observations to the main examination period yielded similar results (data not reported).

Regarding differences in the type of support provided by arm (online supplemental table 3), MSS participants had 13% the odds of SAU participants of using high-intensity UCS support compared with low-intensity support (OR 0.13, 95% CI 0.02 to 0.72, p=0.02), and 22% the odds compared with mid-intensity support (OR 0.22, 95% CI 0.05 to 1.00, p=0.05). There were no statistically significant differences between the use of low- and middle-intensity support (OR 1.71, 95% CI 0.70 to 4.20, p=0.24).

Table 2 shows the self-reported use of mental health resources overall and by arm. Overall, 51% of the students who completed this question reported using at least one of these resources, with many students using more than one resource (median of two resources among those who used them). In both arms, the most frequently used resource was seeing their college supervisor, director of studies or tutor (27% of those who responded to the question). Those who chose the category ‘other resources’ had the chance to explain further. Of the 16 people who chose this category, 13 (MSS=9, SAU=4) mentioned friends, family or loved ones. There are no significant differences between the arms in whether participants used any resources or not (χ^2^=0.36, df=1, p=0.55), the number of resources used (quasi-Poisson regression coefficient=0.03, p=0.87) or in the usage by type of resource (all p values >0.3).

### Other outcomes comparing arms

Table 2 shows the number of participants donating the vouchers offered to recompense them for completion of the 1-year follow-up questionnaires, and the cumulative count of donations throughout the follow-up period. Significantly more MSS participants donated at the 1-year follow-up time point, compared to SAU participants (OR=1.91, 95% CI 1.21 to 3.04, χ^2^=7.88, df=1, p=0.005). Over the course of the year, 101 participants donated once, 106 donated twice and 122 donated thrice. MSS participants donated more times than SAU participants (quasi-Poisson regression coefficient=0.37, p<0.0001).

Table 2 shows participants’ degree of agreement with the statement that their course workload during the past year had been manageable. There were no significant differences between trial arms in whether participants viewed their academic workload as manageable (χ^2^ =4.65, df=4, p=0.33).

Table 2 presents the number of adverse events counted at the 1-year follow-up, and the cumulative count of adverse events throughout the follow-up period. There were fewer adverse events in the MSS arm than in the SAU arm. All of the adverse events in the period between the examination period time point and the 1-year follow-up time point were generated by the monitoring of the CORE-OM risk subscales,^12^ and none of them was considered by the IDMEC as an adverse effect deriving from mindfulness practice. Overall, four people experienced more than one adverse event in the year, and they were all SAU.

### Mindfulness practice effects

**Figure 3:**
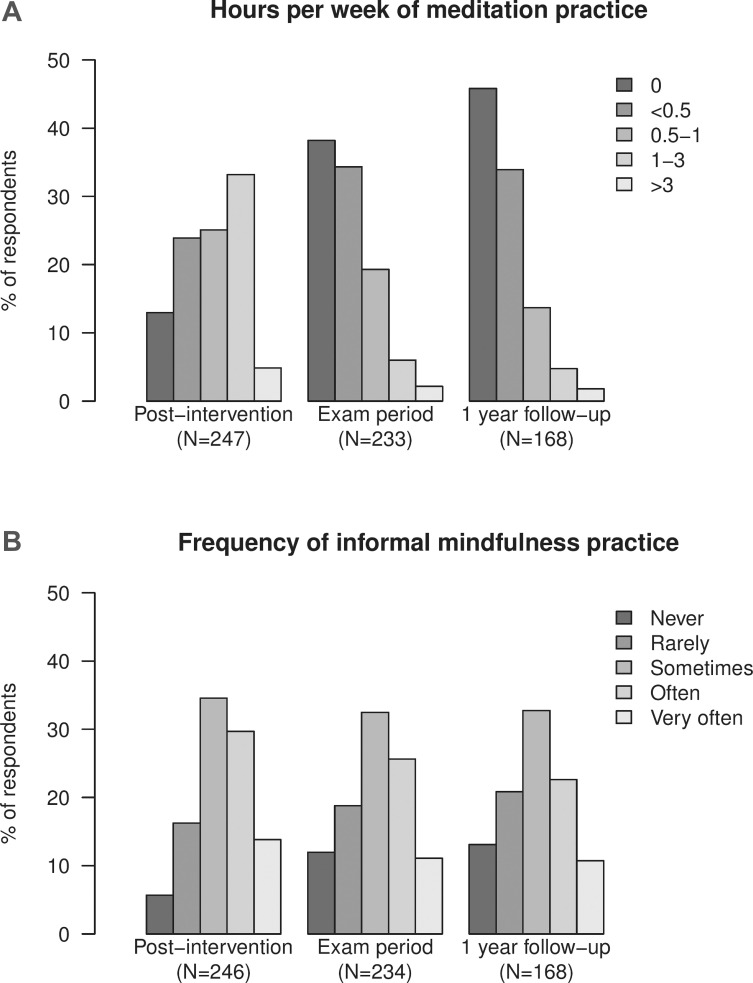
Frequency of formal (A) and informal (B) mindfulness practice at home at each time point.

Figure 3 shows the frequency of formal mindfulness meditation and informal mindfulness exercises respectively at each time point for the MSS participants who answered these questions. Most participants (33%) meditated at home between 1 and 3 hours/week during the MSS course, but meditation dropped sharply later with 38% not having meditated at all between course completion and the examination period, and 46% not having done so after the examination period. However, doing informal mindfulness exercises was more stable, with most participants reporting doing them ‘sometimes’ (35%, 33% and 33% at post intervention, exam period and 1-year follow-up, respectively). After 1 year, at least 33 (11%) SAU participants had practised more than 10 hours of any type of meditation (all of them either mindfulness or vipassana^24^) or done an 8-week mindfulness course.

Having practised formal mindfulness meditation significantly reduced psychological distress at all time points at post-intervention (post-intervention estimate=−0.01, p<0.001; examination period estimate=−0.005, p=0.03; 1-year follow-up estimate=−0.005, p=0.003; model in online supplemental figure 3). Having practised informal mindfulness exercises significantly reduced distress at all time points and with larger effect sizes (post-intervention estimate=−0.08, examination period estimate=−0.09, 1-year follow-up estimate=−0.09, all p values <0.001, model in online supplemental figure 4). Having practised formal or informal mindfulness exercises improved well-being at all time points (formal practice: post-intervention estimate=0.17, p<0.001; examination period estimate=0.12, p=0.001; 1-year follow-up estimate=0.09, p=0.004. Informal practice: post-intervention estimate=1.80, p<0.001; examination period estimate=1.82, p<0.001, 1-year follow-up estimate=1.26, p=0.01).

## DISCUSSION

After 1 year, average distress and well-being levels improved in both trial arms. Multiple factors could account for this: regression to the mean, increasing familiarity with the university environment, recent return from summer holidays or even graduation. Our evidence supports an average beneficial effect of the MSS course on students’ psychological distress and mental well-being that lasts at least a year. The effect seems to be larger at stressful times: the CORE-OM difference between the MSS and the SAU participants corresponded to a moderate effect size during the examination period according to Cohen’s rules of thumb,^13 25^ while after a year this difference was slightly smaller (−0.25 vs −0.22 CORE-OM points). This pattern is consistent with the hypothesis that mindfulness training increases resilience to stress. It also may explain why mindfulness-based programmes are being used in clinical settings, or as indicative preventative interventions for those with subclinical symptoms. Still, universal interventions not explicitly addressing mental health may appeal to those who would otherwise not seek help, as they are less stigmatising.^13^ Small-to-moderate effect sizes are typical of this type of interventions,^26^ which aim to impact by producing small changes in broad sections of the population. The MSS group format makes such large-scale implementation easier and impact swifter. In any case, we only provide evidence on a voluntary student course. Appropriateness, acceptability and effectiveness of incorporating mindfulness training into students’ compulsory curricula are still unclear.^27 28^ Mindfulness courses may not be suitable or engaging for some groups of people. We favour the implementation of the MSS to be offered along with other preventative interventions as part of a wider student well-being strategy.

The MSS course may not impact the subjective experience of managing academic workload^29^ or the frequency of use of mental health support services. However, it may impact the type of mental health support needed in a desirable direction: SAU participants needed more intensive types of UCS support that indicated more severe circumstances, while MSS participants needed types of UCS support that indicated milder severity. MSS participants may have experienced less severe problems, and/or they were more pro-active at asking help. This finding was not evident in the self-reported use of mental health resources—although the latter was only available from approximately half of participants and questions lacked sensitivity in determining support intensity. Economic implications of these results for the UCS are being explored in an economic evaluation currently being conducted.

Participants randomised to the MSS arm have consistently donated more than those allocated to SAU. This may partly be a specific effect of mindfulness training, but it is possible that MSS participants felt more predisposed to donate than SAU participants because they were offered the MSS course, while those in the SAU arm were offered nothing. Therefore, the extra donations may have worked more as a ‘payment for a service’, so more related to a sense of justice than altruism.

Despite MSS course teacher’s advice, very few students continued practising formal mindfulness meditation after the course, although they reported continuing practising mindfulness informally in their everyday life. Formal practice requires dedicated time, while informal practice (eg, washing dishes mindfully) does not; this may explain our results. Our dose–response analyses suggest that mindfulness practice matters: the more participants practised, formally or informally, the more benefit they got. This makes informal practice especially relevant: adherence is good and it still has desirable effects.

### Comparison with existing evidence

Our study confirms previous evidence, derived from smaller and/or lower-quality trials conducted in different settings and countries, that mindfulness courses reduce distress among university students.^10^ Very few studies have looked at longer-term effects among students. One trial followed 288 students up for 6 years and found increased well-being compared with a no-intervention control although only a third of the sample were responsive by then.^30^

Similarly to our findings, Bondolfi *et al* found that following course completion, frequency of informal mindfulness practice remained unchanged over 14 months, whereas the use of formal meditation decreased over time.^31^ A recent systematic review found that participants do on average 64% of the formal practice amount requested during the course, with high variability.^32^ We are unable to calculate such a figure with our data regarding adherence to formal practice during the course, but our results are roughly aligned with it.

We have found beneficial effects to be correlated with mindfulness practice. Agreeing with our findings, a recent systematic review found a small but significant association between formal mindfulness practice during the course and post-intervention outcomes.^32^ Analyses of associations between formal practice after the course and follow-up outcomes are scarce and inconsistent.^33–35^

Very few studies have assessed the frequency and effects of informal mindfulness practice,^33^ in part because of the difficulties in measuring it.^36^ A recent dose–response analysis found that informal practice was associated with improved positive emotions with no association with negative emotions.^37^ Other studies have not found associations.^38 39^ Our finding that those who practice more get more benefit only apply to contexts where beginner mindfulness practitioners practise in their everyday lives, and do not inform about dose–response effects in intensive practice contexts such as meditation retreats. Similarly, they do not inform the quality of the practice (i.e., what/how participants practise). Quality could be a critical factor in determining practice effects,^33^ particularly given the generally low level of support offered to participants once mindfulness courses have concluded.

Recent systematic reviews indicate an effect of mindfulness training on prosocial behaviours, although this may only be true in studies where the meditation teacher was a co-author and the control group was passive.^18 40^ When a meditation course aiming to cultivate empathy was compared with an active control (stretching), the intervention failed to show clear evidence of increased altruism despite increased prosocial reflection.^41^ These support the idea that SAU participants in our trial donated less because of not receiving an intervention.

Our active monitoring system has found no evidence of adverse effects related to self-harm, harm to others or suicidality among MSS participants. However, there are suggestions that subtler adverse effects may go underreported unless asked about specifically^42^—further research is needed.

### Strengths and limitations

This RCT is the largest, to our knowledge, assessing mindfulness training for university students. Its careful design and analysis were prespecified in a publicly registered protocol, which minimises reporting biases. However, it lacked an active control intervention beyond the standard support on offer to students. Therefore, it is not possible from our data to find out to what extent results are influenced by participants’ expectations, peer and teacher support, and other factors unspecific to mindfulness training. However, there are reasons to think that at least part of the effect seen in this trial is specific to mindfulness.^11^ Outcomes were self-reported and participants were not blind to trial arm, meaning that responses may have been indeed influenced by their expectations. Loss to follow-up was considerable, and despite our efforts to collect data, reasons for loss to follow-up are unknown to us. Requesting personal, as well as institutional, email addresses at the start of the study might have helped to mitigate this.

UCS data had no loss to follow-up and were collected from the UCS directly rather than self-reported, making these results highly reliable. However, this was planned as a secondary outcome, and the service intensity subgroup analyses are subject to multiple testing bias.

In contrast to most studies, we measured formal and informal practice. Our analyses of the impact of practice on mental health discard reverse-causality and take into account contamination in the control group. However, they did not compare randomly allocated groups, so they may be subject to residual confounding (eg, those with more time to spare may meditate more and also feel less distressed). In addition, we treated nominal variables as continuous which may contribute bias.

**What is already known on this subject**

- A recent systematic review of trials suggests that, measured shortly after their completion, mindfulness‐based programmes improve university students’ distress and well-being in comparison with passive controls.^10^ More research is needed to assess longer-term effects and mental health service use. Poor trial methodology undermines confidence in review results, highlighting the need for higher-quality trials. How long the effects of a universal intervention to increase resilience to stress last, and whether support services are affected, are key questions for policymakers to plan ahead.

**What this study adds**

- Our study shows that the benefits of the Mindfulness Skills for Students course on students’ psychological distress and mental well-being last at least a year, but that students still use university mental support services at similar rates. We also show that the beneficial effects seem to be larger at stressful times, a pattern that could be interpreted as an increase in students’ resilience to stress. Preventive mindfulness courses are likely to benefit the average university student but should not be seen as a replacement of other mental health support services, for which there will continue to be demand.

## References

[1] NHS Digital Mental health of children and young people in England. 22 Nov 2018.

[2] UK Department for Education Participation rates in higher education: academic years 2006/2007–2017/2018 (provisional). 2019 Available https://assets.publishing.service.gov.uk/government/uploads/system/uploads/attachment_data/file/843542/Publication_HEIPR1718.pdf

[3] Faculty of Public Health and Mental Health Foundation *Better mental health for all: a public health approach to mental health improvement*. London, 2016.

[4] Auerbach RP, Mortier P, Bruffaerts R, et al. WHO world mental health surveys international college student project: prevalence and distribution of mental disorders. *J Abnorm Psychol* 2018 10.1037/abn0000362 PMC6193834 30211576

[5] Bewick B, Koutsopoulou G, Miles J, et al. Changes in undergraduate students’ psychological well‐being as they progress through university. *Stud. Higher Ed* 2010;35:633–45. 10.1080/03075070903216643

[6] Barkham M, Broglia E, Dufour G, et al. Towards an evidence-base for student wellbeing and mental health: definitions, developmental transitions and data sets. *Couns Psycho Res* 2019;19:351–7. 10.1002/capr.12227

[7] Thorley C Not by degrees: improving student mental health in the UK’s universities. 2017 Available https://www.ippr.org/research/publications/not-by-degrees

[8] Barnes N, Hattan P, Black DS, et al. An examination of mindfulness-based programs in US medical schools. *Mindfulness* 2017;8:489–94. 10.1007/s12671-016-0623-8

[9] Mindfulness All-Party Parliamentary Group Mindful Nation UK Report. The Mindfulness Initiative. 2015 Available http://themindfulnessinitiative.org.uk/images/reports/Mindfulness-APPG-Report_Mindful-Nation-UK_Oct2015.pdf (accessed 10 07 2017)

[10] Dawson AF, Brown WW, Anderson J, et al. Mindfulness-based interventions for university students: a systematic review and meta-analysis of randomized controlled trials. *Appl Psycho Health Well-Being* 2019 10.1111/aphw.12188 31743957

[11] Goyal M, Singh S, Sibinga EMS, et al. Meditation programs for psychological stress and well-being: a systematic review and meta-analysis. *JAMA Intern Med* 2014;174:357–68. 10.1001/jamainternmed.2013.13018 24395196 PMC4142584

[12] Galante J, Dufour G, Benton A, et al. Protocol for the Mindful Student Study: a randomised controlled trial of the provision of a mindfulness intervention to support university students’ well-being and resilience to stress. *BMJ Open* 2016;6 10.1136/bmjopen-2016-012300 PMC5129000 28186934

[13] Galante J, Dufour G, Vainre M, et al. A mindfulness-based intervention to increase resilience to stress in university students (the Mindful Student Study): a pragmatic randomised controlled trial. *The Lancet Public Health* 2018;3:e72–e81. 10.1016/S2468-2667(17)30231-1 29422189 PMC5813792

[14] Williams M, Penman D *Mindfulness: a practical guide to finding peace in a frantic world*. London, UK: Hachette, 2011.

[15] Connell J, Barkham M, Mellor-Clark J The effectiveness of UK student counselling services: an analysis using the CORE system. *Br J Guid Counc* 2008;36:1–18. 10.1080/03069880701715655

[16] Core System Group CORE system user manual. Available http://www.coreims.co.uk/index.html (accessed 15 09 2015)

[17] Stewart-Brown S, Janmohamed K *Warwick-Edinburgh mental well-being scale user guide. Version 1*. University of Warwick, 2008.

[18] Donald JN, Sahdra BK, Van Zanden B, et al. Does your mindfulness benefit others? A systematic review and meta-analysis of the link between mindfulness and prosocial behaviour. *Br J Psychol* 2018 10.1111/bjop.12338 30094812

[19] R Core Team *R: A language and environment for statistical computing*. Vienna, Austria: R Foundation for Statistical Computing, 2017.

[20] Byrne BM *Structural equation modeling with AMOS*. London: Routledge, 2010.

[21] Mirman D *Growth curve analysis and visualization using R*. Florida: CRC Press, 2014.

[22] Muthén L, Muthén BO *Mplus user’s guide*. 8th ed Los Angeles, CA: Muthén & Muthén, 1998–2019.

[23] Warwick Medical School WEMWBS: 14-item vs 7-item scale. 2019 Available https://warwick.ac.uk/fac/sci/med/research/platform/wemwbs/about/wemwbsvsswemwbs/ (accessed 11 12 2019)

[24] Chiesa A Vipassana meditation: systematic review of current evidence. *J Altern Complement Med* 2010;16:37–46. 10.1089/acm.2009.0362 20055558

[25] Cohen J *Statistical power analysis for the behavioral sciences*. 2nd edn. Hillsdale, NJ: Lawrence Erlbaum Associates, 1988.

[26] Public Health England Decision making in public health: using number needed to treat (NNT) to determine intervention effectiveness. 2014 Available http://www.nwph.net/Publications/NNT_FINAL.pdf (accessed 10 07 2017)

[27] Stewart-Brown S, Cader M, Walker T, et al. Experiences with a universal mindfulness and well-being programme at a UK medical school. *Health Educ* 2018;118:304–19. 10.1108/HE-10-2017-0053

[28] Brown CG *Debating yoga and mindfulness in public schools reforming secular education or reestablishing religion?* Chapel Hill: The University of North Carolina Press, 2019.

[29] Bóo SJM, Childs‐Fegredo J, Cooney S, et al. A follow‐up study to a randomised control trial to investigate the perceived impact of mindfulness on academic performance in university students. *Couns Psychother Res* 2019;20:286–301. 10.1002/capr.12282

[30] de Vibe M, Solhaug I, Rosenvinge JH, et al. Six-year positive effects of a mindfulness-based intervention on mindfulness, coping and well-being in medical and psychology students; results from a randomized controlled trial. *PLoS One* 2018;13:e0196053 10.1371/journal.pone.0196053 PMC5916495 29689081

[31] Bondolfi G, Jermann F, der Linden MV, et al. Depression relapse prophylaxis with mindfulness-based cognitive therapy: replication and extension in the Swiss health care system. *J Affect Disord* 2010;122:224–31. 10.1016/j.jad.2009.07.007 19666195 PMC2866251

[32] Parsons CE, Crane C, Parsons LJ, et al. Home practice in mindfulness-based cognitive therapy and mindfulness-based stress reduction: a systematic review and meta-analysis of participants’ mindfulness practice and its association with outcomes. *Behav Res Ther* 2017;95:29–41. 10.1016/j.brat.2017.05.004 28527330 PMC5501725

[33] Lloyd A, White R, Eames C, et al. The utility of home-practice in mindfulness-based group interventions: a systematic review. *Mindfulness* 2018;9:673–92. 10.1007/s12671-017-0813-z 29875880 PMC5968057

[34] Solhaug I, de Vibe M, Friborg O, et al. Long-term mental health effects of mindfulness training: a 4-year follow-up study. *Mindfulness* 2019;10:1661–72. 10.1007/s12671-019-01100-2

[35] Madson L, Klug B, Stimatze T, et al. Effectiveness of mindfulness-based stress reduction in a community sample over 2 years. *Ann Clin Psychiatry* 2018;30:52–60. 29373618

[36] Segal Z, Dimidjian S, Vanderkruik R, et al. A maturing mindfulness-based cognitive therapy reflects on two critical issues. *Curr Opin Psychol* 2019;28:218–22. 10.1016/j.copsyc.2019.01.015 30798103 PMC6661222

[37] Fredrickson BL, Arizmendi C, Van Cappellen P, et al. Do contemplative moments matter? Effects of informal meditation on emotions and perceived social integration. *Mindfulness* 2019;10:1915–25. 10.1007/s12671-019-01154-2

[38] Crane C, Crane RS, Eames C, et al. The effects of amount of home meditation practice in mindfulness based cognitive therapy on hazard of relapse to depression in the staying well after depression trial. *Behav Res Ther* 2014;63:17–24. 10.1016/j.brat.2014.08.015 25261599 PMC4271738

[39] Hawley LL, Schwartz D, Bieling PJ, et al. Mindfulness practice, rumination and clinical outcome in mindfulness-based treatment. *Cognit Ther Res* 2014;38:1–9. 10.1007/s10608-013-9586-4

[40] Kreplin U, Farias M, Brazil IA The limited prosocial effects of meditation: a systematic review and meta-analysis. *Sci Rep* 2018;8:2403 10.1038/s41598-018-20299-z 29402955 PMC5799363

[41] Galante J, Bekkers MJ, Mitchell C, et al. Loving-kindness meditation effects on well-being and altruism: a mixed-methods online RCT. *Appl Psychol Health Well Being* 2016;8:322–50. 10.1111/aphw.12074 27333950

[42] Van Dam NT, van Vugt MK, Vago DR, et al. Mind the hype: a critical evaluation and prescriptive agenda for research on mindfulness and meditation. *Perspect Psychol Sci* 2017 10.1177/1745691617709589. PMC5758421 29016274

