# Supplementary material for: Effectiveness of providing university students with a mindfulness-based intervention to increase resilience to stress: 1-year follow-up of a pragmatic randomised controlled trial
Source: J Epidemiol Community Health. 2020 Sep 10;75(2):151–60. doi: 10.1136/jech-2020-214390 (PMC7116569; doi:10.1136/jech-2020-214390)
Supplement: Supplementary data [file jech-2020-214390supp001.pdf]

Effectiveness of providing university students with a mindfulness-based intervention to increase resilience to stress: one-year follow-up of a pragmatic randomised controlled trial

Julieta Galante, Jan Stochl, Géraldine Dufour, Maris Vainre, Adam P Wagner, Peter B Jones

Supplementary Materials

Supplementary Methods: Pre-processing practice data

Mindfulness practice data required pre-processing for inclusion within models. Questions had frequency Likert scales transformed to numeric values. For formal meditation practice, the MSS group was asked how much they had practiced in an average week, while the SAU group was asked about the total amount of meditation within the specified period. Response items consisted of hour ranges (e.g. "Between 0.5 and 1h per week"). In order to estimate a figure representing hours of practice we took a mid-value of the range for control and intervention options, then for the intervention participants who were asked how much they had practiced on an average week, we multiplied the weekly value by the number of weeks in the period. For the post-intervention meditation practice calculation in the intervention group, we also added half an hour of meditation for each course session attended (only added in the dose-response models).

In the model assessing the effect of formal mindfulness practice, we included all trial participants because we have meditation data for the control group as well as the intervention group. The model assessing informal mindfulness practice only included intervention participants since there is no data collected on this in the SAU group.

Supplementary Tables

Supplemental Table 1. Outcome measures reported in this publication with the time points at which data for each was collected.

Type of data collected	Baseline	Post-intervention	Exam period	One-year follow-up
Demographics & prior meditation	X			
Attendance at mindfulness courses (MSS arm)		X		
Mindfulness course homework (MSS arm)		X		
Practised after the course (MSS arm)			X	X
Practised meditation elsewhere (SAU arm)		X	X	X
Psychological distress	X	X	X	X
Wellbeing	X	X	X	X
Donations		X	X	X
Use of mental health services				X
Use of University Counselling Service				X
Workload manageable				X

Abbreviations: MSS=mindfulness skills for students; SAU=support as usual

Supplemental Table 2. UCS services according to the intensity of the support they provide

Intensity level	UCS resource
1	Assertiveness Group<br>MPhil Group<br>Returners Group<br>Returners - Anxiety<br>Returners - Self Compassion<br>Returners - Time Management<br>Workshop - Anxiety<br>Workshop - CBT for self-help<br>Workshop - Exam Preparation<br>Workshop - Food and mood<br>Workshop - Panic attacks<br>Workshop - Procrastination<br>Workshop - Self-compassion<br>Workshop - Sleep<br>Workshop - Social anxiety
2	Counselling<br>Assessment<br>Bereavement Group<br>Client Contact by Phone or Email<br>Managing Mood Group<br>Perfectionism Group<br>Postgraduate Group<br>Self-esteem Group<br>Undergraduate Group
3	Mental Health Advisor - Assessment<br>Mental Health Advisor - Ongoing

Supplemental Table 3. Use of University Counselling Service: differences between intervention and control groups by levels of support (hierarchical multinomial logit model).

UCS support type	Estimate	SE	P value	OR	OR 95%CI
High vs low	-2.05	0.87	0.02	0.13	0.02 to 0.72
High vs middle	-1.51	0.77	0.05	0.22	0.05 to 1.00
Low vs middle	0.54	0.46	0.24	1.71	0.70 to 4.20

Abbreviations: 95%CI= 95% confidence interval; OR=odds ratio; UCS= University Counselling Service.

Supplementary Figures

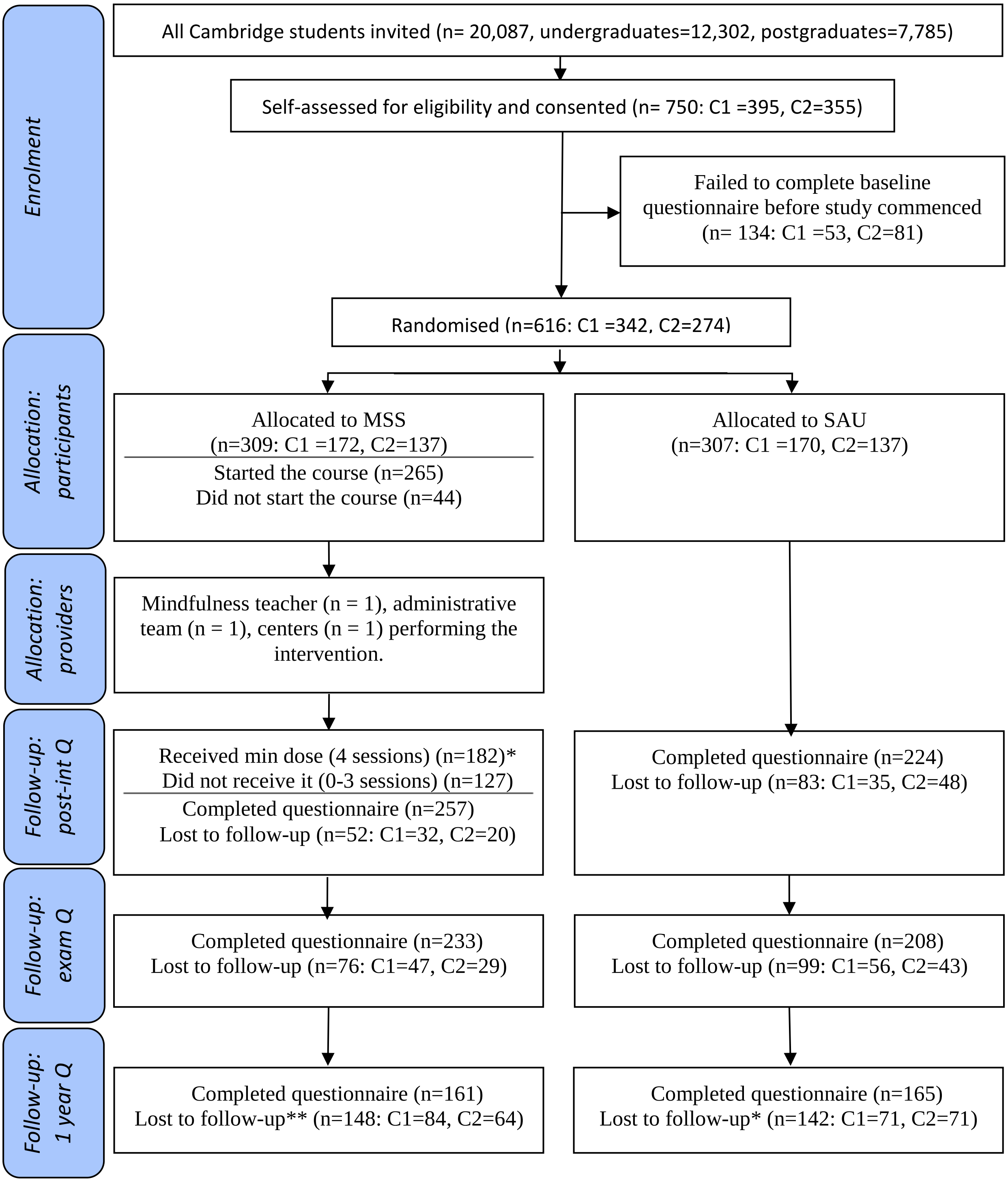

Supplemental Figure 1. CONSORT 2010 flow diagram. See text and tables for number of participants included in each analysis. * A minimum dose of four sessions is typically suggested in mindfulness research (Gu et al. 2015). ** No reasons were given for non-completion. Abbreviations: C1= study cohort 1; C2= study cohort 2; min= minimum; Q= questionnaire; post-int= post intervention, MSS=mindfulness skills for students; SAU=support as usual.

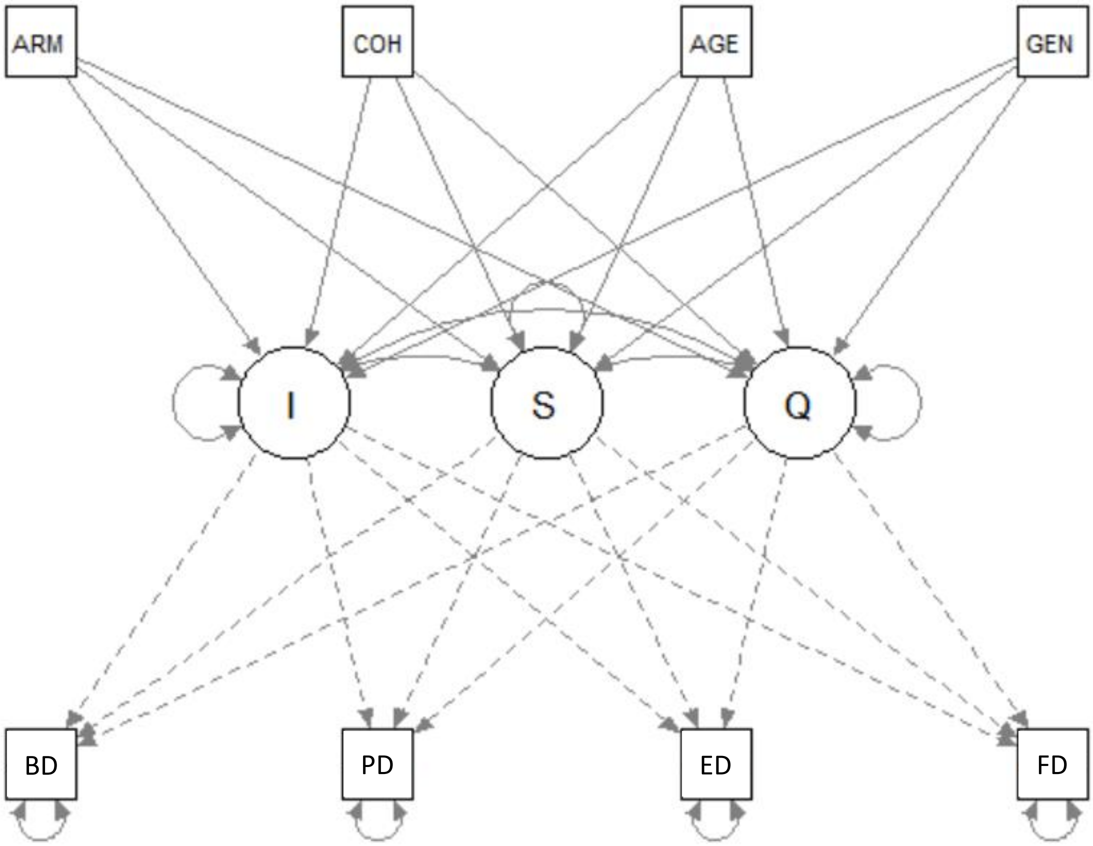

Supplemental Figure 2. Growth mixture model conceptual path diagram for outcome psychological distress. Dashed lines are fixed parameters whereas solid lines are estimated in the model. See Byrne 2010 or Mirman 2014 (references in the main text) for further detail on such models. Abbreviations: COH=cohort; GEN=gender; I=intercept; S=slope; Q= quadratic term; BD=baseline distress (CORE-OM); PD=post-intervention distress (CORE-OM); ED=exam-term distress (CORE-OM); FD=follow-up distress (CORE-OM).

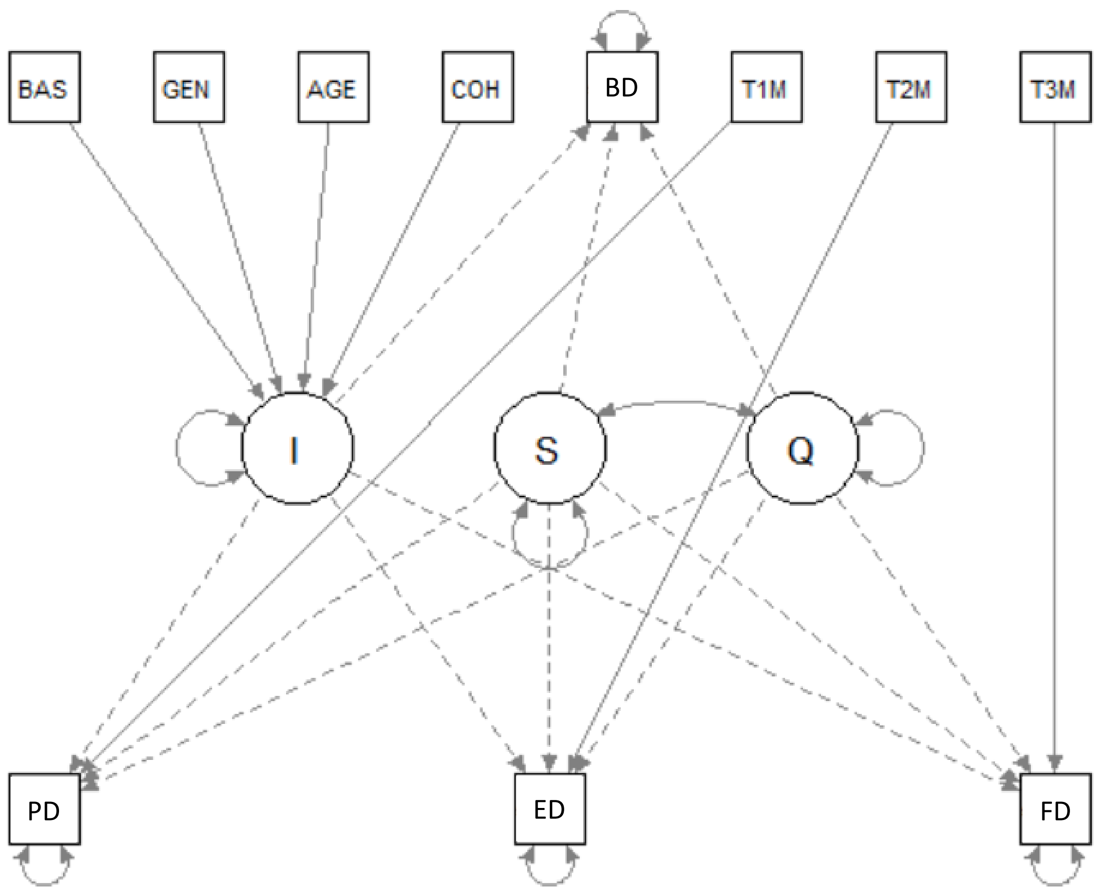

Supplemental Figure 3. Growth mixture model conceptual path diagram testing the influence of meditation practice (time-varying co-variates) on psychological distress. Dashed lines are fixed parameters whereas solid lines are estimated in the model. See Byrne 2010 or Mirman 2014 (references in the main text) for further detail on such models. Abbreviations: BAS=baseline (prior) meditation experience, COH=cohort; GEN=gender; I=intercept; S=slope; Q=curve; BD=baseline distress (CORE-OM); T1M=meditation measured at post-intervention; T2M=meditation measured during exam term; T3M=meditation measured at one-year follow-up; PD=post-intervention distress (CORE-OM); ED=exam-term distress (CORE-OM); FD=follow-up distress (CORE-OM).

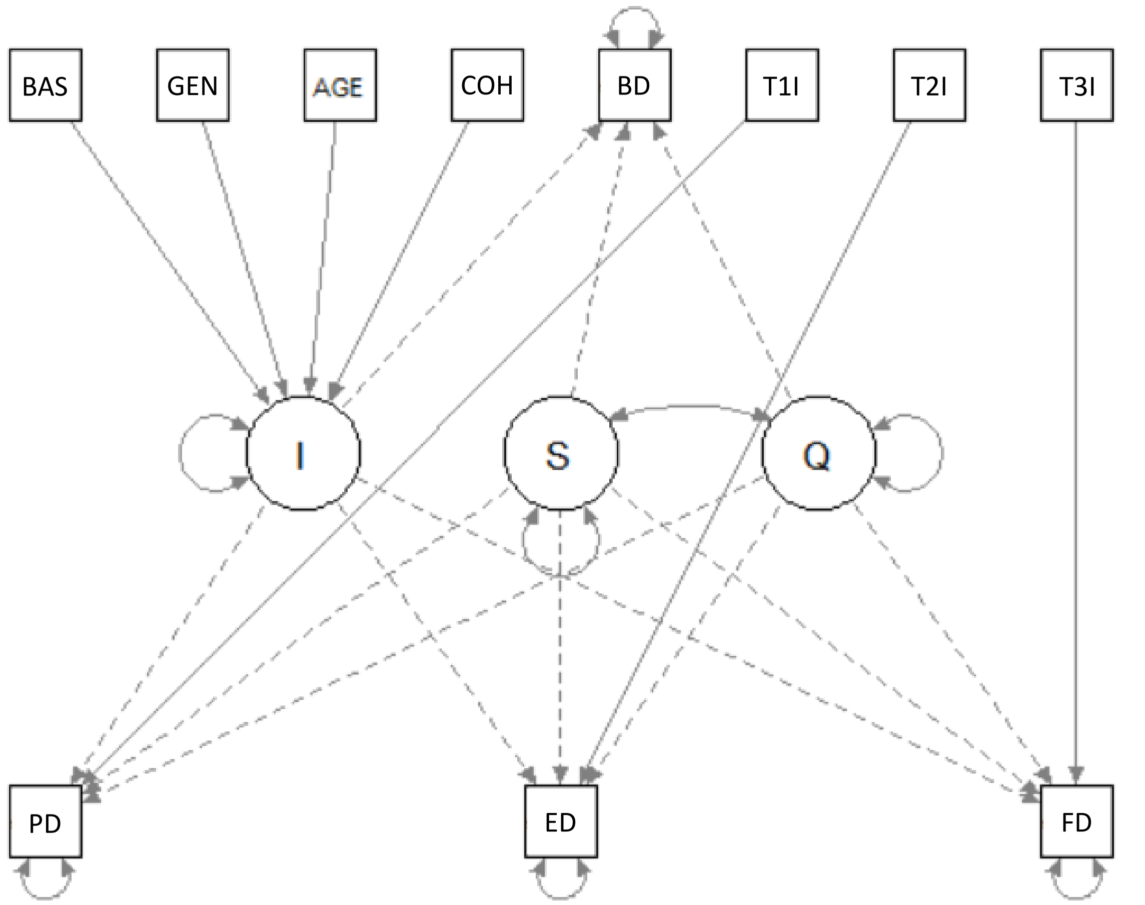

Supplemental Figure 4. Growth mixture model conceptual path diagram testing the influence of informal mindfulness practice (time-varying co-variates) on psychological distress. See Byrne 2010 or Mirman 2014 (references in the main text) for further detail on such models. Abbreviations: BAS=baseline (prior) meditation experience, COH=cohort; GEN=gender; I=intercept; S=slope; Q=curve; BD=baseline distress (CORE-OM); T1I=informal mindfulness measured at post-intervention; T2M=informal mindfulness measured during exam term; T3M=informal mindfulness measured at one-year follow-up; PD=post-intervention distress (CORE-OM); ED=exam-term distress (CORE-OM); FD=follow-up distress (CORE-OM).
